# The Effects of Dental Hygiene Instruments on Streptococcus sanguinis Adhesion to Titanium Implant Abutments

**DOI:** 10.7759/cureus.66605

**Published:** 2024-08-10

**Authors:** Nadzirah Muhamad Nasir, Haslina Taib, Mohamad Arif Awang Nawi, Akram Hassan

**Affiliations:** 1 School of Dental Sciences, Universiti Sains Malaysia, Kota Bharu, MYS

**Keywords:** implant maintenance, streptococcus sanguinis, bacterial adhesion, hygiene instruments, dental implants

## Abstract

Introduction: Maintenance of dental implant with different hygiene methods or instruments may cause a surface alteration. It directly affects bacterial colonization and adhesion on titanium implant surfaces that result in peri-implant diseases. This study aimed to compare the *Streptococcus sanguinis (S. sanguinis) *adhesion on titanium implant abutments after instrumentation with a rubber cup with pumice and erbium, chromium-doped: yttrium, scandium, gallium, and garnet (Er, Cr: YSGG) laser using scanning electron microscope (SEM) observation and colony-forming unit (CFU) measurement.

Methods: Twenty-one MegaGen titanium implant abutments were randomly distributed into three groups. Seven abutments were respectively selected for the control/untreated (C) group, while the other two groups were treated with rubber cups with pumice (P) and Er, Cr: YSGG laser (L). All samples were cultured with *S*. *sanguinis* for bacterial colonization and adhesion. One sample for each group was selected for SEM observation, while the other samples were prepared for CFU calculation.

Results: For SEM results, at 2,000× magnification, machining marks were intact in the C group, roughened in the L group, and smoothened in the P group. At 5,000× and 10,000× magnifications, moderate colonies of *S. sanguinis* were revealed in C and L groups, while sparse bacterial colonies were detected in the P group. However, for CFU results, statistical analysis showed no significant value (p>0.05) comparing all three groups.

Conclusion: P instrumentation revealed a lesser amount of *S. sanguinis* adhesion in SEM photographs, but no statistical significance of CFU results was noted for all three groups.

## Introduction

Dental implants have been used for a few decades to replace missing tooth or teeth and even a part of a prosthesis to the completely edentulous jaw [1]. A systematic review reported that the osseointegrated implants have a high success rate (94.6%) and minimal marginal bone resorption (1.3 mm) with a follow-up period of at least 10 years [2]. However, the implant placement may also expose the patients to biological complications [3] as well as mechanical complications [4] that can lead to implant failure.

Peri-implant diseases such as peri-implant mucositis and peri-implantitis are the biological complications of dental implants caused by infections initiated by bacterial biofilm and subsequently inflammation of the soft tissues and bone surrounding implants [5,6]. Thus, prevention of these peri-implant diseases can be achieved by regular maintenance care of dental implants from at-home implant care by the patients and dental healthcare professionals [7] that aims to reduce plaque formation on dental implant surfaces.

For implant maintenance by dental professionals, different hygiene instruments have been used and reported in previous studies to prevent peri-implant diseases such as metal and non-metal curettes, sonic and ultrasonic scalers, air polishing devices, rubber cups with polishing pastes, locally applied chemotherapeutics, subgingival irrigation and laser irradiation (diode, erbium-doped: yttrium, aluminum, and garnet (Er: YAG) and erbium, chromium-doped: yttrium, scandium, gallium, and garnet (Er, Cr: YSGG)) [8-11]. Unfortunately, a study by Louropoulou et al. [12] proved that these methods have various surface profile modifications and surface roughness that may affect newly formed biofilm on dental implant smooth and rough surfaces. However, these implant cleaning methods should aim to be less biofilm-retentive and consequently reduce the de-novo formation of the biofilm [12].

The transmucosal portion of the implant surface that is exposed to the intraoral environment is smooth. On the contrary, plaque and calculus may accumulate more easily on surfaces with grooves, scratches, and other surface modifications caused by polishing equipment. Therefore, maintaining clean smooth surfaces with precautions to protect the implant surfaces is necessary for the prevention of peri-implant diseases.

*Streptococcus sanguinis* (*S. sanguinis*)* *is one of the *Streptococci* species that predominantly colonizes the implant surfaces once they have been exposed to the oral environment. These early colonizers develop an auspicious condition for the late colonizers to adhere to implant surfaces [13]. Thus, the implant surfaces should be prepared ideally to reduce the number of early colonizers and consequently reduce the adhesion of late colonizers and the formation of plaque biofilm [14].

For this study, two hygiene instruments which were the conventional rubber cup with pumice and more advanced erbium, chromium-doped: yttrium, scandium, gallium, and garnet (Er, Cr: YSGG) laser irradiation were chosen for comparison to investigate the effects of these methods toward *S. sanguinis *bacterial adhesion on titanium implant abutment surfaces through surface topography under scanning electron microscope (SEM) and colony-forming units (CFUs) measurement. Therefore, the objectives of this study were to describe the* S. sanguinis* bacterial adhesion of titanium-implant abutment surface topography in groups of untreated (control), treated with rubber cup with pumice and Er: Cr, YSGG laser and also measure the amounts of CFU of these bacteria in these three groups.

## Materials and methods

Hygiene instruments

Twenty-one MegaGen ST (MegaGen Implant Company South Korea) titanium implant abutments (n=21) were used in the study. Each of the abutments was mounted on the dental stone. Out of 21, seven abutments (n=7) were randomly selected for each group: untreated/control (C) group, rubber cup with pumice (P) group, and Er, Cr: YSGG laser (L) group.

Saliva collection and sterility

A healthy donor has formally provided written informed consent. Unstimulated saliva was naturally collected for five days, with each taken daily between 7:30 am and 9:30 am. Patients were asked to rinse with water to remove food residue and rest at least 10 minutes after rinsing before saliva collection. The saliva samples were collected in 15-ml sterile tubes and were preserved in a cool flask with a container before being transported to the laboratory. The samples were frozen at -20°C until 45 ml was collected.

The saliva samples were pooled in a 50-ml sterile centrifuge tube and centrifuged at 2,500 revolutions per minute (rpm) for 30 minutes. Subsequently, the supernatant was pasteurized for 30 minutes at 60°C in a water bath to inactivate endogenous enzymes and eventually was re-centrifuged in the 50-ml sterile centrifuge tube at 2,500 revolutions per minute (rpm) for 30 minutes and stored at -20°C. The efficacy of pasteurization was evaluated by plating 0.1 ml brain-heart infusion (BHI), and the absence of bacterial growth was observed after 72 hours to confirm the sterility of the saliva samples.

*Streptococcus sanguinis *culture

*S. sanguinis *bacteria cells from standard strain ATCC 10556 Thermo Scientific USA were cultured in blood agar plates using an aseptic technique and the streak plate method, where the individual cells were diluted by spreading them over the surface of the agar plate. The plate was incubated at 37°C in a candle jar and the bacterial colonies were observed after 24 hours. The bacterial cells were later transferred and sub-cultured on the blood agar for subsequent tests. 

Saliva coating for pellicle formation

The titanium implant abutments were autoclaved for 20 minutes at 121°C. Subsequently, they were placed in a sterile 24-well polystyrene cell-culture plate containing 1 ml of saliva and kept at 37°C for four hours to allow salivary pellicle formation.

Inoculum preparation and optical density

After four hours, inoculums were prepared by harvesting the standard reference strain *S. sanguinis* cells ATCC 10556 (Thermo Scientific USA) from the previously cultured blood agar plate in the brain-heart infusion (BHI) broth. These bacterial cells were suspended in the BHI broth, and turbidity was adjusted to an optical density (OD)_630_ 0.15 (~10^6^ colony-forming units (CFUs/ ml) using a spectrophotometer (Shimadzu Japan).

Adhesion assay

Subsequently, the saliva was aspirated from each well-containing titanium implant abutment in each well and replaced with 0.5 ml brain-heart infusion (BHI) broth (Oxoid UK) and 0.5 ml saliva. Each well was inoculated with 0.1 ml of previously prepared inoculum suspension. These titanium implant abutments were incubated at 37°C in a candle jar for 16 hours.

Serial dilutions

After 16 hours, six abutments of each group (n=18) were washed in sterile distilled water to remove unattached cells and inserted in microtubes containing 1 ml of sterile peptone water. The microtubes were vigorously vortexed for two minutes to free the bacteria attached to the surface of each specimen and to disperse the bacterial cells. These suspensions underwent serial dilutions (10^-1^ to 10^-5^). The first dilution was done by taking 0.1 ml with a pipette from each microtube into 0.9 ml of BHI broth to produce 1 ml of 10^-1^ diluted solution. The process was repeated four times to achieve successful serial dilutions. The bacterial cells were inoculated in brain-heart infusion (Hi media India) agar plates (three plates for each dilution) and incubated at 37°C for 48 hours.

Colony-forming unit (CFU) measurement for bacterial adhesion

The colony-forming unit (CFU) in this study was the number of colonies of *S. sanguinis* that grow on a brain-heart infusion (BHI) agar plate. The tests were done in triplicate, and the CFUs of *S. sanguinis* on the BHI agar plates for each dilution were measured manually. The 10^-4^ dilution exhibited the highest number of plates with a statistically significant range of 30 to 300 colonies [15]. Plates with 30 to 300 colonies for each sample were averaged and selected for data analysis. The plates with colonies count of more than 300 were noted as too numerous to count (TNTC), while plates with colonies of less than 30 were noted as too few to count (TFTC).

Scanning electron microscopy (SEM) for bacterial topography on titanium implant abutments

One abutment from the respective group (n=3) was selected for observation of bacterial topographies under a scanning electron microscope (SEM). After 16 hours of incubation and colonization of *S. sanguinis*, the abutments were washed in phosphate-buffered saline (PBS) a few times. Next, the samples were fixed with 4% paraformaldehyde in 0.1 M phosphate buffer (pH 7.4) for two hours at room temperature. They were incubated with 8% paraformaldehyde for two days at 4°C. Subsequently, they were dehydrated in graded ethanol (30%, 50%, 70%, 80%, 90%, 100%, 100%) for 10 minutes of each solution. Lastly, the samples were incubated in hexamethyldisilazane (HMDS) solution for 10 minutes before being air-dried in a desiccator for five minutes.

Eventually, all three abutments were gold sputtered with Leica EM SCD 005 gold sputter coater. The flat surface of the prepared portion of the specimens was mounted facing the stub. The transmucosal portion (2 mm × 3 mm convex surface) was standardized for scanning at three points. Photographs at 2,000×, 5,000×, 10,000×, and 20,000× magnifications were captured as the representative areas for the samples to describe the adhesion and colonization of *S. sanguinis* bacteria.

## Results

SEM for *S. sanguinis* bacterial topography on titanium implant abutments

**Figure 1:**
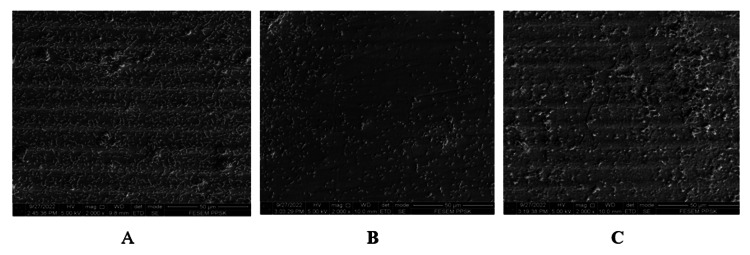
S. sanguinis adhesion on titanium implant abutments photographed at 2,000× magnification. (A) Control. (B) Rubber cup with pumice. (C) Er, Cr: YSGG laser. Er, Cr: YSGG: erbium, chromium-doped: yttrium, scandium, gallium and garnet.

*S. sanguinis* bacterial colonization and adhesion on three titanium implant abutments (one for each group) were scanned and photographed under SEM by 2,000×, 5,000×, and 10,000× magnifications. At 2,000× magnifications (Figures 1A-1C), the machining marks of untreated/control titanium implant abutment surfaces were still intact. On the other hand, the abutment that was treated with a rubber cup and pumice showed a smoothened abutment surface with a loss of machining marks. The abutment that was treated with Er, Cr: YSGG laser irradiation also showed intact machining marks but with a roughened surface and the presence of melting effects compared to untreated/control abutment.

**Figure 2:**
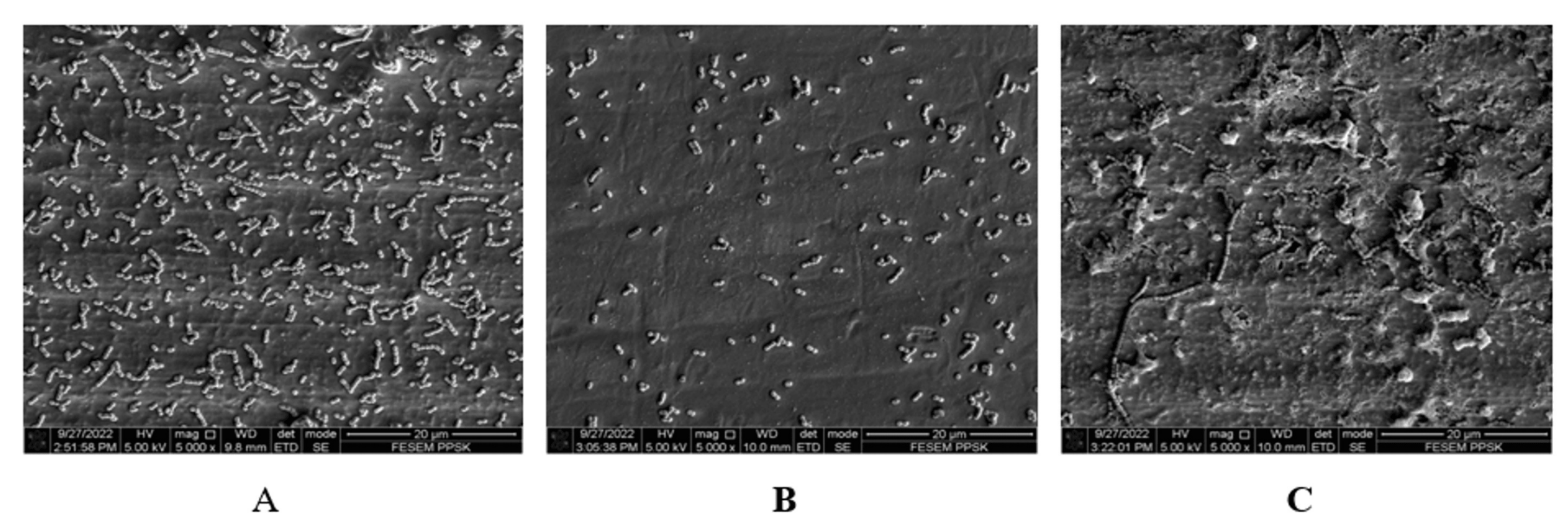
S. sanguinis adhesion on titanium implant abutments photographed at 5,000× magnification. (A) Control. (B) Rubber cup with pumice. (C) Er, Cr: YSGG laser. Er, Cr: YSGG: erbium, chromium-doped: yttrium, scandium, gallium, and garnet.

At 5,000× magnification (Figures 2A-2C), moderate colonies of *S. sanguinis* were noted in untreated/control abutment and abutment that was treated with Er, Cr: YSGG laser. On the contrary, sparse bacterial colonies were noted on the titanium implant abutment treated with a rubber cup and pumice.

**Figure 3:**
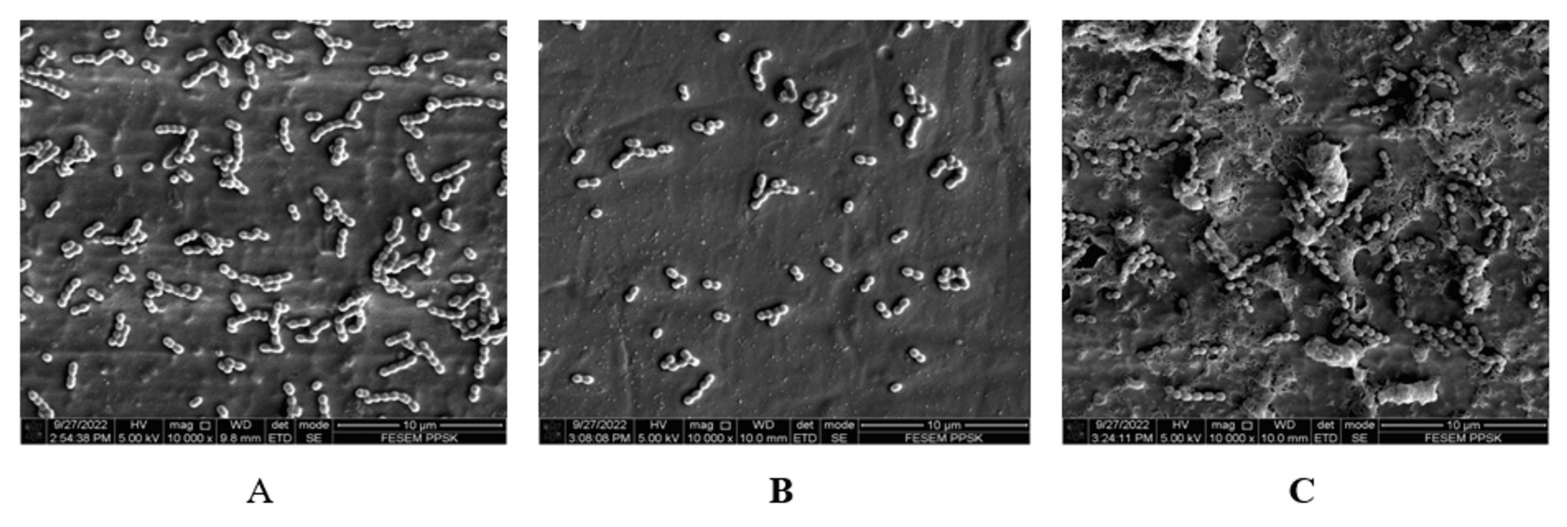
S. sanguinis adhesion on titanium implant abutments photographed at 10,000× magnification. (A) Control. (B) Rubber cup with pumice. (C) Er, Cr: YSGG laser. Er, Cr: YSGG: erbium, chromium-doped: yttrium, scandium, gallium, and garnet.

At 10,000× magnification (Figures 3A-3C), untreated/control titanium implant abutment showed mostly multilayer chains of *S. sanguinis* bacterial cells. Besides, the abutment treated with a rubber cup and pumice revealed mostly a monolayer chain of bacterial cells. Furthermore, the abutment treated with Er, Cr: YSGG laser showed a multilayer chain of bacterial cells.

Colony-forming units (CFUs)

**Figure 4:**
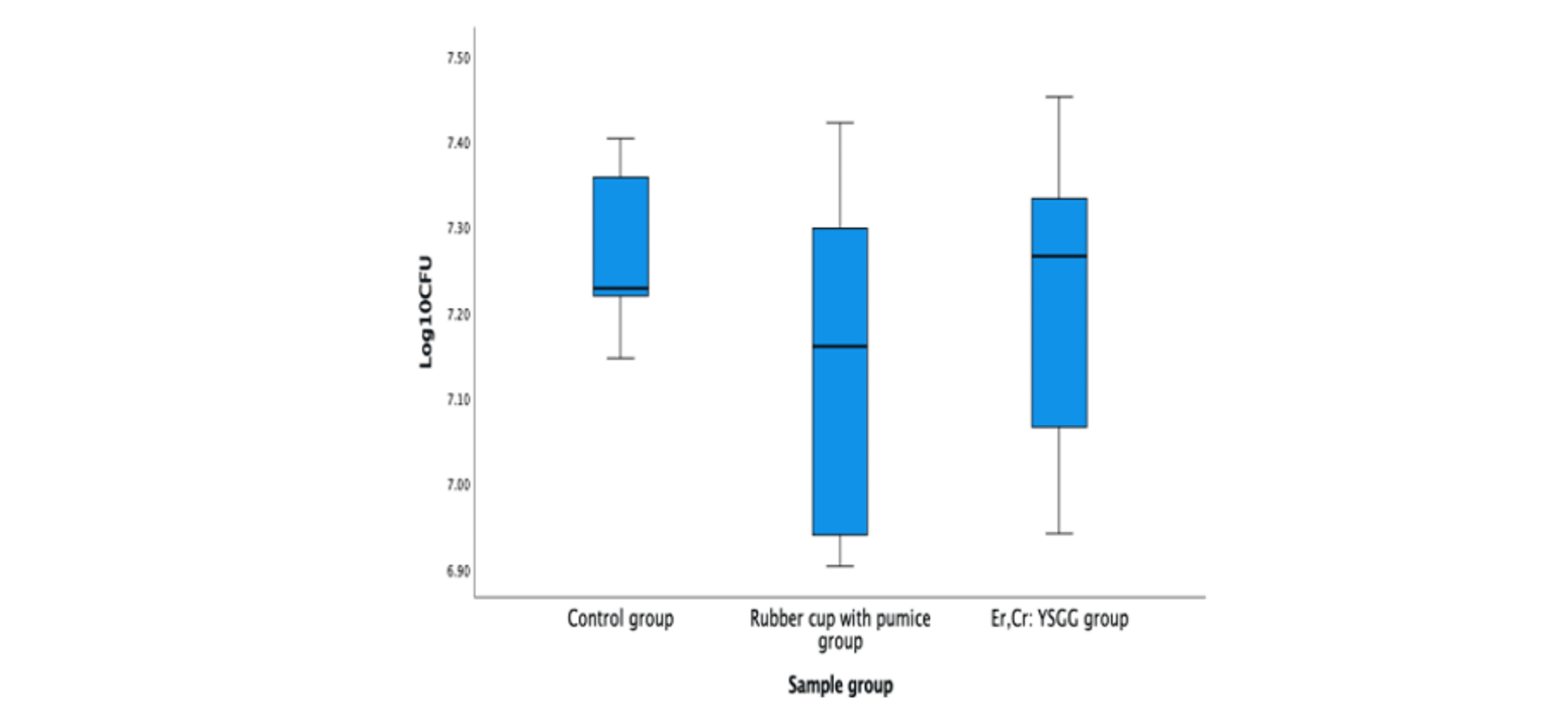
The mean amounts of log10 CFU/ml S. sanguinis on titanium implant abutments for three different groups. Er, Cr: YSGG: erbium, chromium-doped: yttrium, scandium, gallium, and garnet; CFU: colony-forming unit.

Based on descriptive statistics (Table 1 and Figure 4), the mean amounts of log_10_ CFU/ml *S. sanguinis* were different among groups where the lowest mean was in the rubber cup with pumice group followed by Er, Cr: YSGG laser group and untreated/control group. One-way ANOVA parametric test (Table 2) was used to compare means of log_10_ CFU/ml *S. sanguinis *adhesion on titanium implant abutments within and between these three groups.

**Table 1:** Descriptive statistics for the means of log10 CFU/ml of S. sanguinis on titanium implant abutments for the three groups. Er, Cr: YSGG: erbium, chromium-doped: yttrium, scandium, gallium, and garnet; CFU: colony-forming unit.

Group	n	Mean	SD	Minimum	Maximum
Control	6	7.26	0.10	7.15	7.40
Rubber cup with pumice	6	7.14	0.20	6.90	7.42
Er, Cr: YSGG laser	6	7.22	0.19	6.94	7.45

**Table 2:** One-way ANOVA comparing mean amounts of log10 CFU/ml of the three groups. p-value significant at <0.05 at a 95% confidence interval. CFU: colony-forming unit.

Group	Sum of squares	df	Mean square	F	Sig.
Between groups	0.042	2	0.021	0.735	0.496
Within groups	0.426	15	0.028		
Total	0.467	17			

However, the one-way ANOVA result proved that the means of log_10_ CFU/ml of *S. sanguinis* adhesion were not statistically significant F (2,15) = 0.735, p = 0.496 between and within all three groups. This result provided strong evidence that there was no significant difference in bacterial adhesion on titanium implant abutments among untreated/control, treated with a rubber cup with pumice, and treated with Er, Cr: YSGG laser.

## Discussion

*S. sanguinis* adhesion on titanium implant abutments following various applications of hygiene instruments

The finding of this study was congruous with a previous study by McCollum et al. [16] reported that when utilized with light intermittent pressure, the rubber cup containing pumice flour offered the smoothest polished abutment surface. However, the implant abutments might gouge or become rounded at the abutment-prosthesis interface when the rubber cup is applied with excessive force. A systematic review by Louropoulou et al. [12] suggested that rubber cups, whether they contain paste or not, are "implant-safe" since they essentially do not harm smooth implant surfaces. However, the SEM result of this study showed that the titanium implant abutments that have been treated with rubber cups and pumice produced smooth surfaces with non-intact machining marks. The original irregular scratches on the surfaces were obliterated.

The findings of this study revealed low *S. sanguinis* bacterial adherence in the rubber cup-containing pumice group compared to the control/untreated group and Er, Cr: YSGG laser group. Therefore, those titanium implant abutments treated with rubber cups with pumice showed sparse distribution of bacterial colonies with a monolayer of *S. sanguinis* bacterial cell chains. This result was in accordance with the study by Di Salle et al. [17] whereby reduced bacterial adhesion was demonstrated in titanium discs that were treated with a rubber cup using an abrasive paste.

Meanwhile, Er, Cr: YSGG laser irradiation was reported in SEM results in previous literature as an effective implant decontamination method without producing any surface modification [11,18,19]. Nevertheless, the SEM result for Er, Cr: YSGG laser group in this study noted to produce surface roughness with melting effects, and this may be due to inconsistency of laser parameters setting in these studies such as power, distance, and time of irradiation.

On the other hand, the findings of the other two groups, the control/untreated group and Er, Cr: YSGG laser group, revealed a moderate distribution of bacterial colonies with a multilayer of *S. sanguinis *bacterial cell chains. The result was consistent in a nearly similar study by Duarte et al. [9] that reported moderate *S. sanguinis* colonization in both untreated and treated smooth titanium implant discs. However, the former study used Er: YAG laser irradiation as one of the treatment modes. In addition, limited data on bacterial adhesion as the effects of Er, Cr: YSGG laser irradiation was noted in the literature since most studies focused on the efficacy of this laser in removing biofilm. Besides the heterogeneities for direct comparison with previous studies, the results that were produced from SEM findings in this present study should be cautiously interpreted since they were too subjective and may reveal false conclusions since only one sample was taken for observation for each group.

Colony-forming units on titanium implant abutments following various applications of hygiene instruments

*S. sanguinis* is a commensal bacterium that is abundantly found in oral biofilm. This bacterium is associated with oral health but also has interaction with caries and periodontitis-associated pathogens besides forming biofilm of different implant surfaces [20]. *S. sanguinis* was frequently used in adherence model studies because it was crucial for the development of bacterial plaque [21]. In addition, this type of bacteria had superior adhesion to saliva-coated surfaces compared to other microbes, even though it was not connected to the sites present with active bone loss in periodontitis or peri-implantitis [21,22]. Based on these reasons, *S. sanguinis* bacteria was chosen in this study.

This present study demonstrated that the highest mean log_10_ CFU values of the *S. sanguinis* bacteria were noted in the control/untreated group compared to treated groups (rubber cup with pumice and Er, Cr: YSGG laser irradiation). This finding proved that the titanium implant surfaces need to be treated for maintenance. On the other hand, statistical analysis of this study produced non-significant results of the mean log_10_ CFU values of *S. sanguinis* bacterial adhesion when compared within and between three hygiene instrumentation groups. The results revealed that no best single hygiene instrument could be advocated to the patient.

In agreement with this result, a previous study by Duarte et al. [9] concluded that the level of *S. sanguinis* adhesion was similar in untreated and treated smooth titanium discs despite the differences in titanium surface profiles captured in SEM photographs. Besides, a study by Schmidt et al. [23] gave consistent results with this present study when they concluded that although there were some differences in the mean log_10_ CFU values among various instrumentation groups, the result of the effects on the bacterial adhesion after instrumentation was not proved to be significant.

In comparison to research that measured the amount of bacterial adherence using CFU, more studies subjectively described bacterial adhesion as an effect following hygiene devices using SEM. Thus, insufficient data was collected for comparison on the amounts of bacterial CFU. Although there were multiple studies done on the effects of these implant hygiene instruments on surface modifications and bacterial colonization, no treatment modalities have been regarded as the gold standard for implant decontamination since each of the methods has its advantages and disadvantages. Furthermore, many other aspects including patients’ factors and the feasibility of the equipment should be looked into consideration before clinical recommendations to the patients.

Recommendations

Future research should consider other methods of polishing or hygiene instruments such as curettes, ultrasonic devices, chemotherapeutic agents, and other types of lasers for better comparative study. In addition, dental implants from other systems and surface treatments such as plasma-sprayed, chemical-etched, or sand-blasted titanium surfaces should be used for further investigation. For microbiology studies, multiple bacteria can be used in future research since biofilm formation is diverse and exists as multispecies bacterial communities. To mimic real clinical conditions, an in-vivo clinical study should be conducted to evaluate the effect of surface changes on biofilm accumulation.

## Conclusions

Surface topographies of *S. sanguinis* bacterial adhesion revealed moderate colonies with multilayer bacterial cells chains both in control/untreated and Er, Cr: YSGG laser groups, while sparse colonies with monolayer chains of bacterial cells noted in rubber cup and pumice group. The amounts of *S. sanguinis* CFU bacterial adhesion on titanium implant abutments revealed the lowest mean of log_10_ CFU values noted in the rubber cup and pumice group followed by Er, Cr: YSGG laser and control/untreated group. However, statistical analysis revealed no significant difference in the mean of log_10_ CFU values noted within and between the three groups.
